# Simple Approaches to Minimally-Instrumented, Microfluidic-Based Point-of-Care Nucleic Acid Amplification Tests

**DOI:** 10.3390/bios8010017

**Published:** 2018-02-26

**Authors:** Michael G. Mauk, Jinzhao Song, Changchun Liu, Haim H. Bau

**Affiliations:** Mechanical Engineering and Applied Mechanics (MEAM), School of Engineering and Applied Science, University of Pennsylvania, Towne Building, 220 33rd Street, Philadelphia, PA 19104, USA; songjinz@seas.upenn.edu (J.S.); lchangc@seas.upenn.edu (C.L.); bau@seas.upenn.edu (H.H.B.)

**Keywords:** microfluidics, nucleic acid amplification test, molecular diagnostics, LAMP (loop mediated amplification), RPA, NAAT, lab on a chip

## Abstract

Designs and applications of microfluidics-based devices for molecular diagnostics (Nucleic Acid Amplification Tests, NAATs) in infectious disease testing are reviewed, with emphasis on minimally instrumented, point-of-care (POC) tests for resource-limited settings. Microfluidic cartridges (‘chips’) that combine solid-phase nucleic acid extraction; isothermal enzymatic nucleic acid amplification; pre-stored, paraffin-encapsulated lyophilized reagents; and real-time or endpoint optical detection are described. These chips can be used with a companion module for separating plasma from blood through a combined sedimentation-filtration effect. Three reporter types: Fluorescence, colorimetric dyes, and bioluminescence; and a new paradigm for end-point detection based on a diffusion-reaction column are compared. Multiplexing (parallel amplification and detection of multiple targets) is demonstrated. Low-cost detection and added functionality (data analysis, control, communication) can be realized using a cellphone platform with the chip. Some related and similar-purposed approaches by others are surveyed.

## 1. Introduction and Scope of Review

Microfluidics ‘lab-on-a-chip’ (LOC) technologies enable clinical diagnostics outside of laboratories, and in particular, at the point-of-care (POC). POC diagnostics devices perform in vitro assays of disease-associated biomarkers in clinical specimens such as blood, urine, or saliva [1,2,3,4,5,6,7,8,9,10,11,12,13,14,15,16,17]. Potential benefits of microfluidic diagnostics include short (<60 min) test times; reduced reagent consumption; and ease of use (allowing minimally-trained laymen and non-technical healthcare providers to perform relatively sophisticated diagnostics); thereby facilitating essential tests in resource-poor settings that lack clinical laboratory facilities and other infrastructure. While POC testing addresses the immediate needs of developing or underdeveloped areas of the world, POC devices can also help close gaps in healthcare in all counties by fostering distributed, decentralized and mobile healthcare in venues such as doctors and dentists offices, rural clinics, pharmacies, school infirmaries, and at home with over-the counter test devices. Of particular interest are POC tests based on detection of pathogen-specific nucleic acids due to their high sensitivity and specificity. POC technology can provide sustainable and appropriate medical diagnostics for currently underserved populations. The World Health Organization offers the following criteria (under the rubric ASSURED, see for example [18]) for POC infectious disease test technology for resource-limited settings: Affordable, Sensitive (few false negatives), Specific (few false positives), User-friendly, Rapid, Equipment-free, and Delivered to those with need. Table 1 lists some desirable features and performance objectives for POC devices, especially targeted for routine use in resource-limited areas of the world.

A ‘lab-on-a-chip’ diagnostics system is based on a disposable, single-use, credit card-sized, plastic cassette (cartridge or ‘chip’) that hosts a microfluidic network for sample processing and analysis. The chip is used in combination with supporting instrumentation such as a small, portable desktop or palm-sized unit to provide controlled heating, valves’ and fluids’ actuation, and detection. Recent trends in POC technology include minimally-instrumented formats, such as battery-powered or electricity-free diagnostics devices, and utilization of smartphones (mobile communication device with an operating system to run software applications) for detection, analysis, communication, GPS, and other functions. For example, and as discussed herein as an illustrative case, a cartridge with a single chamber combining sample processing and sequence-specific nucleic acid detection, and with pre-stored reagents and integral pouches for liquid storage, serves as comparatively simple diagnostics device, requiring minimal flow control and supporting instrumentation.

Although LOC systems can be used for a wide variety of applications, including cancer and genetic disease diagnostics, food and water testing, bioterrorism surveillance, and laboratory research, we focus this review on specific implementations for infectious disease ‘molecular’ (nucleic acid based) diagnostics of viral and bacterial pathogens. Of main interest are microfluidic devices that integrate sample processing and nucleic acid amplification, with applications to minimally-instrumented devices for resource-limited settings. Such LOC devices perform multiple processing steps for extracting nucleic acids from heterogeneous clinical specimens, amplifying the nucleic acids to enable detection by optical means. We first discuss the advantages and requirements of nucleic acid amplification tests for POC diagnostics, and review design options for microfluidic implementation of each processing step, as well as their integration in a microfluidic cartridge. Options for parallel amplification reactor arrays for multiplex (multiple nucleic acid detection, e.g., from different pathogens), cellphone-based detection, and modules for plasma extraction from whole blood are also described.

## 2. Background: Medical Diagnostics and POC Testing

Broadly, medical diagnosis [19] utilizes one or a combination of: (1) Microscopic examination of organisms in clinical specimens, e.g., observation of stained blood smears for malaria parasites; (2) medical imaging, e.g., chest X-rays for signs of TB; (3) culture and detection of organisms in, e.g., blood or stool; (4) immunoassays of pathogen-specific antigens or antibodies in clinical samples; and (5) detection of pathogen-specific DNA or RNA in specimens, enhanced with a nucleic acid amplification step such as PCR (polymerase chain reaction). Such assays with DNA or RNA as a target analyte are termed Nucleic Acid Amplification Tests (NAATs) or molecular diagnostics. The first three methods may require excessive time in acute infectious disease situations, and further, their use in resource-limited settings may be constrained by scarce expertise and the need for expensive equipment. Immunoassays are attractive for POC applications because they can utilize unprocessed or crude samples such as whole blood or urine, and can sometimes provide test results in minutes, but usually in a few hours. Lateral flow strip immunoassays, as represented by the familiar home pregnancy test, HIV saliva antibody tests (OraQuik™, OraSure Technologies, Inc., Bethlehem, PA, USA), and drugs of abuse tests, are prominent examples of rapid, low-cost, easy-to-use, commercially-available point-of-care diagnostics [20]. Because NAATs amplify target nucleic acids several million-fold or more, they are generally at least a thousand times more sensitive than immunoassays. However, NAATs conventionally require sample processing and relatively expensive laboratory-based equipment [21]. Microfluidics offers excellent prospects to make POC molecular diagnostics as simple to use and almost as inexpensive as lateral flow strip immunoassays.

**Figure 1:**
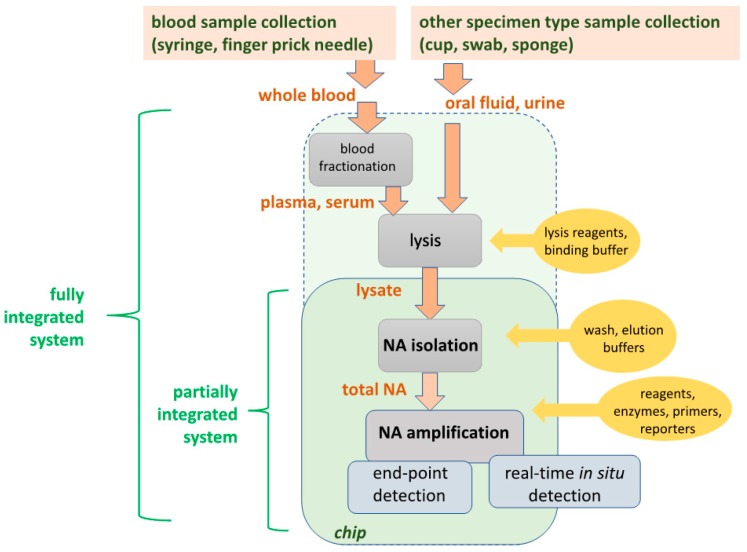
Processing steps (‘unit operations’) of Nucleic Acid Amplification Tests (NAATs) molecular diagnostics. Plasma from whole blood samples, or raw oral fluid or urine, is lysed to release nucleic acids (DNA or RNA) from virus or cellular pathogens. The soluble nucleic acid is isolated in a purified, concentrated form. Pathogen-specific nucleic acid is enzymatically amplified using PCR (polymerase chain reaction) or isothermal amplification methods. The amplification products (a positive test result) are detected either after amplification (end-point detection) by color dyes sensitive to DNA, or in real-time (during amplification) by, e.g., fluorescence due to intercalating dyes, or bioluminescent reporters coupled to the amplification reaction. Reagents (enzymes, primers, reporters) and buffers are required at the steps indicated.

Molecular diagnostics tests typically comprise sequential steps (‘unit operations’) to: (1) Lyse cells or virus; (2) extract, purify and concentrate total DNA or RNA from the lysate, and (3) amplify specific nucleic acid target sequences to facilitate their detection by optical or electrochemical means, either as a positive/negative endpoint test or a real-time monitoring of amplification product generation for quantifying the target template in the sample. Figure 1 shows a process flow for molecular diagnostics. For blood tests, plasma or serum is separated from whole blood before lysis. For oral fluid (‘saliva’), urine, or other sample types, raw sample is directly subjected to lysis. Partial integration, combining NA isolation (extraction, purification, and concentration of total NA), enzymatic NA amplification, and amplicon detection on a single chip, is well established, as described in the review. Full integration, including plasma isolation and lysis, are also feasible, but optimum design approaches are application dependent, i.e., specific to type of pathogen (virus, Gram+ or Gram− bacteria, parasite), sample type (blood, urine, oral fluid), sample volume (5 to 1000 μL). Table 2 shows various design options for implementing each unit operation. POC microfluidic applications predominantly employ a combination of chemical lysis, solid-phase nucleic acid isolation, isothermal amplification, and optical detection. The process of Figure 1 is implemented in the laboratory with spin column kits based on porous silica as a nucleic acid capture phase and chaotropic salt solutions as a binding agent [22,23], and requires a centrifuge and a benchtop thermal cycler for PCR. Such solid-phase extraction can also be readily implemented in a microfluidic format. Alternatively, after lysis, specific nucleic acids can be captured by hybridization [24,25], followed by release and detection or optionally amplification/detection [26]. In another option, bacteria or viruses can be immunocaptured or fractionated by physical means, followed by lysis, nucleic acid isolation, and amplification/detection [27,28]. The extraction and NA isolation step removes substances that inhibit amplification [29]. Microfluidic implementations of solid-phase extraction can use various binding phase materials (silica, alumina, polymers, porous membranes, microstructures, packed beds, and beads) [30,31]. Chips for NA isolation using silica-based binding phases [32,33,34,35,36,37] nanoporous alumina [38], polymeric monoliths [39], and chitosan [40,41] have been described, often integrated with amplification and detection. In addition to solid-phase extraction, other methods based on immiscible phases [42,43], surface functionalization and surface charging are feasible [44]. Microfluidic nucleic acid solid-phase extraction with silica binding phases can be enhanced by adding carrier RNA to the lysate [45]. For whole blood samples, plasma containing virus and bacteria is first separated from the sample using a centrifuge or other means, thus removing red blood ‘cells’ that would otherwise tend to clog the device as well as hemoglobin and other substances which inhibit or interfere with downstream processing. In summary, NAATs offer high sensitivity and specificity, but require considerable sample processing (compared to immunoassays, and cytology. The diagnostics test can be usefully viewed as a sequence of unit operations, and simplified operation is achieved by their seamless integration on a single microfluidic cartridge (‘chip’).

## 3. Microfluidic Molecular Diagnostics

**Figure 2:**
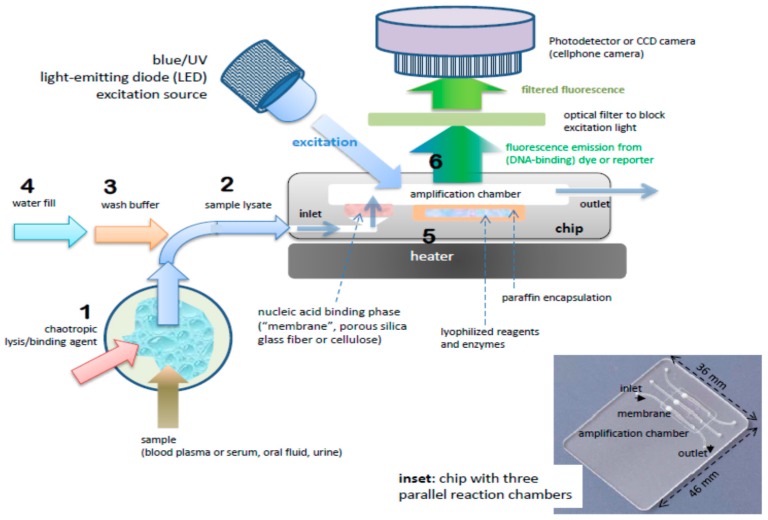
Process steps and (cross-section) schematic of chip for nucleic acid amplification test (NAAT). Chip has one or more flow-through chambers for isothermal amplification and includes a filter-like, flow-porous nucleic acid binding phase (e.g., silica glass fiber or cellulose) and is pre-loaded with paraffin-encapsulated amplification reagents (lyophilized polymerase, primers, fluorescence reporter DNA-intercalating, dyes, and other components). Operational steps: (1) sample is mixed off-chip with lysis/binding reagent buffer (containing e.g., chaotropic agent such as guanidinium HCl) that lyses virus and cells and promotes nucleic acid adsorption to binding media, e.g., silica glass fiber or cellulose (‘membrane’), (2) sample (~100 µL) is injected into chip with pipette or syringe, (3) ethanol-based, high-salt buffer (~100 µL) is injected into chip to wash the membrane (keeping most of the captured nucleic acid adsorbed to the membrane, (4) chamber (25 to 50 µL volume) is filled with water and sealed with tape, (5) chip is heated to amplification temperature (~65 °C) using a small (~1 Watt) electric-heater. The heating melts the paraffin encapsulation, releasing and reconstituting the reagents, (6) the amplification reaction is excited with a blue or UV LED, such that the DNA intercalating dye generates a fluorescence signal proportional to the amount of DNA amplicon produced. The fluorescence is measured by filtering the excitation light and detection with a photodetector of CCD camera, such as provided by a mounted cellphone.

**Figure 3:**
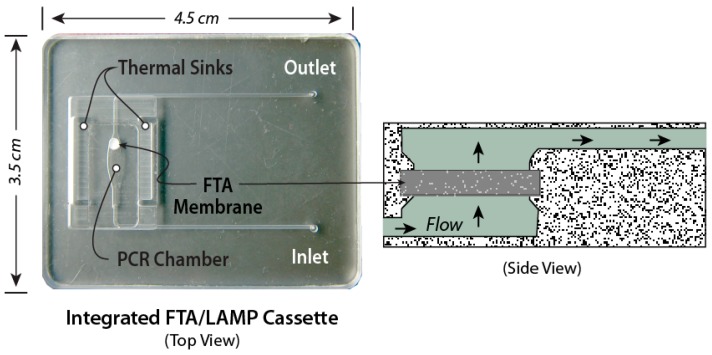
Microfluidic molecular diagnostics chip made as bonded polycarbonate laminate. Amplification (“PCR” or isothermal) chamber volume is 25 μL. Inlet channel has a porous ‘membrane’ disc (~1 mm thick and 2 to 3 mm in diameter) comprised of Whatman FTA™ cellulose or silica glass fiber. Inflow of sample lysate (lysing and nucleic acid binding agent, e.g., guanidium HCl) is filtered through the membrane, capturing nucleic acids from the sample. Relatively large lysate volumes of several hundred microliters or more can be injected into the chip in a “flow-through” filtration mode. The membrane is washed with ethanol:water and backfilled amplification reagent mixture or simply water when the reagents are pre-stored in the chamber. The inlet and outlet ports are sealed with tape. Nucleic acids immobilized on membrane are desorbed in backfilling and/or during subsequent heating, providing template for amplification.

The benchtop molecular diagnostics protocol can be adapted to a microfluidic format [46,47]. Figure 2 shows a slightly schematized example of a chip-based system, featuring an amplification reaction chamber (~25 μL) and a flow-through nucleic acid capture membrane at the inlet of the amplification chamber. The clear plastic chip (Figure 3) is placed on a temperature-controlled stage which includes a light source (light-emitting diode, LED or smartphone flashlight), an optical filter, and detector/camera (possibly smartphone camera) for optical measurements.

**Figure 4:**
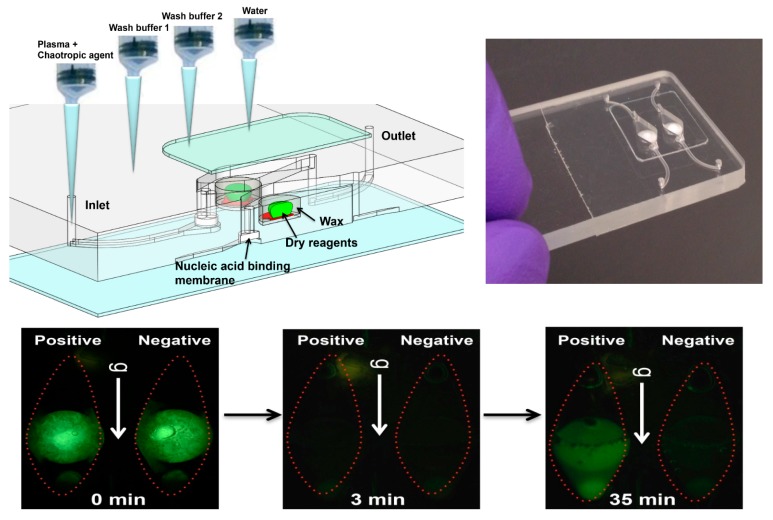
Chip with pre-stored, paraffin-encapsulated, lyophilized amplification reagents (polymerase, primers, nucleotides, magnesium and buffer components, reporter dyes, e.g., Eva Green™ DNA-intercalating fluorescent dye, and enhancers such as betaine and BSA). **Top left:** CAD (computer-aided design) drawing showing cross-section of chamber in forefront and second chamber (including inlet and outlet channels). Chips arrayed with ten or more such parallel chambers are feasible, allowing multiplex testing, or separate chambers for various controls and calibration. Chip is operated with a succession of pipetting steps to add sample (e.g., plasma mixed with chaotropic lysing/binding agent), followed by one or two wash steps, and backfilling the chamber with water; **Top right:** Photo of chip; **Bottom:** Series of top-view photographs of the chips showing fluorescence from two chambers at three stages of amplification: positive (left chamber) and no template negative control (right chamber). Initial green fluorescence (0 min) is due to fluorescence and reflection from solid wax. At 3 min, no signal. At 35 min, positive chamber shows fluorescence while negative chamber remains dark. From [60].

A sample (20 to 500 μL) is mixed with an equal volume of chaotropic salt (6M Guanidium HCl) solution, optionally supplemented with other lysing agents such as proteinase K, lysosome, and detergents (SDS), that lyses cells and viruses and solubilizes nucleic acids. This lysate is then injected into the chip and filtered through the membrane, a porous plug of silica glass fiber or cellulose 1 to 3 mm in diameter and 1 mm in thickness. The chaotropic salt promotes preferential adsorption of nucleic acids to the membrane. Importantly, the method allows one to decouple sample volume from reaction volume and use large sample volume for high sensitivity. The captured nucleic acids immobilized on the membrane are washed with ~200 μL of ethanol/water (50%:50%) solution to remove proteins and other cellular debris. The chamber, which contains pre-stored lyophilized amplification reagents, including enzymes, primers, and reporter dyes, encapsulated in paraffin, is filled with water, the chip inlets/outlets are sealed with tape, and the chip is heated. On heating, the paraffin melts (melting point ≈ 60 °C) just-in-time, releasing the reagents and initiating the amplification reaction (see Figure 4). The solid paraffin encapsulant protects the amplification reagents from dissolution during the sample loading and wash steps, and provides a ‘hot start’ preventing non-specific priming at non-optimal annealing temperatures during the heat-up step [48]. The fluorescence signal, whose emission intensity is proportional to the amount of DNA produced by the amplification reaction, is monitored in real time with a detector or CCD camera. In the simplest version of the chip, the sample loading, wash, and fill steps can be done manually with a pipette. Alternatively, blister packs or pouches containing water and buffer solutions can be attached (with fluid connection) to chip and actuated by finger, or mechanical or electromechanical actuators to affect the wash and filling steps [49,50,51,52,53,54,55]. Similar approaches include chips with integrated flexible polymer films [56] or foils [57]. More elaborate diagnostics chips with micropumps, microvalves, and micromixers have also been described [58,59].

The amplification reaction volume is an important design consideration. Large sample volumes are needed to detect sparse analytes, such as in HIV viral load diagnostics that may need to measure viral RNA at levels as low as 10 copies per mL of plasma. However, there is incentive to use a much smaller amplification reaction volume in order to economize reagents, facilitate rapid temperature control, and improve contrast of optical signals over background noise. In fact, enzyme costs, which scale with reaction volume, can be a substantial (~50%) fraction of total chip cost. Amplification reaction volumes of 10 to 50 μL are typical in chip-based systems, and are in the same range as conventional benchtop protocols.

A few reports describe adding small volumes of raw sample (e.g., 2 μL whole blood) to amplification reaction volumes of 25 to 50 μL. A larger proportion of unprocessed sample in the reaction would exhibit impurity effects, due for example, to the hemoglobin in blood which inhibits polymerase [61,62]. Test protocols based on small volumes of crude sample necessarily translate to poor LODs. Accordingly, molecular diagnostics almost always involves sample preparation to isolate, purify, and concentrate the target nucleic acid for amplification. The solid-phase extraction process described above can concentrate nucleic acid from plasma, saliva, or urine by a factor of ~100, such that when the nucleic acid is concentrated and eluted into a total reaction volume of ~25 μL, an amplification reaction LOD of 10 copies per reaction will correspond to a detection limit of 10 copies per mL of sample. This sensitivity is typically on par with laboratory-based HIV viral load tests. An alternative POC concentration step based dialysis of plasma has also been demonstrated [63]. To summarize, the ‘backend’ process flow steps (see Figure 1), i.e., NA isolation, amplification, and detection, in combination with pre-stored, paraffin-encapsulated, lyophilzied reagents and pouch liquid reservoirs for buffer solutions, can be implemented in a single-chamber process, greatly simplifying flow control and operation.

## 4. Isothermal Nucleic Acid Amplification

In the past, molecular diagnostics relied almost exclusively on the polymerase chain reaction (PCR, thermal cycling) for enzymatic amplification of target sequences [64]. PCR requires comparatively sophisticated instrumentation to achieve precise (±0.5 °C) and rapid (>10 °C/s) thermal cycling. In addition, the relatively high temperature peaks (~100 °C) necessitate good sealing of the reactor to prevent evaporation. Instrumentation for point of care molecular diagnostics includes heating, temperature regulation, and optical detection [65,66,67,68]. More recently, ‘isothermal’ methods, i.e., amplification reactions maintained at a constant incubation temperature, have been developed and offer considerable simplification compared to PCR with respect to temperature control and heating/cooling and better tolerance of impurities than PCR, and are finding increasing use in POC diagnostics. These isothermal methods include LAMP (Loop-mediated AMPlification), RPA (Recombinase Polymerase Amplification), HDA (Helicase Dependent Amplification), NASBA (Nucleic Acid Sequence Based Amplification), RCA (Rolling Circle Amplification), and SDA (Strand Displacement Amplification). Their application to POC diagnostics has been reviewed and compared on various criteria including sensitivity, specificity and ease of implementation [69,70,71,72,73,74,75]. LAMP is probably the most frequently described isothermal PCR alternative in POC microfluidics applications, and is the main focus in this review. Lower reaction temperatures (37 to 65 °C) also reduce evaporation and formation of air bubbles, sealing problems, and energy consumption in heating. Moreover, in many applications, LAMP appears to be less sensitive to inhibitors than PCR [76,77], which is attributed to the higher tolerance of *BSt* polymerase in LAMP compared to *Taq* DNA polymerase in PCR for inhibitors commonly found in clinical specimens [78]. Blood anticoagulants can be problematic for amplification [79,80] and the NA isolation step described earlier alleviates this problem. In addition, novel LAMP enzymes include both reverse transcriptase and strand displacement activities, enabling amplification of both DNA and RNA targets in the same process, circumventing the need for a separate reverse transcription reaction for RNA targets [81]. However, compared to PCR, LAMP requires more complex primer design (four to six primers, instead of two for PCR). Most of the isothermal methods are proprietary, and so may require licensing for commercialization. In addition, constant-temperature amplification can be more difficult to quantify since the simple near doubling of template with each cycle assumed for PCR [82] does not apply to isothermal methods. To summarize, isothermal NA amplification methods, such as LAMP, offer numerous advantages for POC application, especially with regard to minimal instrumentation.

## 5. Chip Fabrication

**Figure 5:**
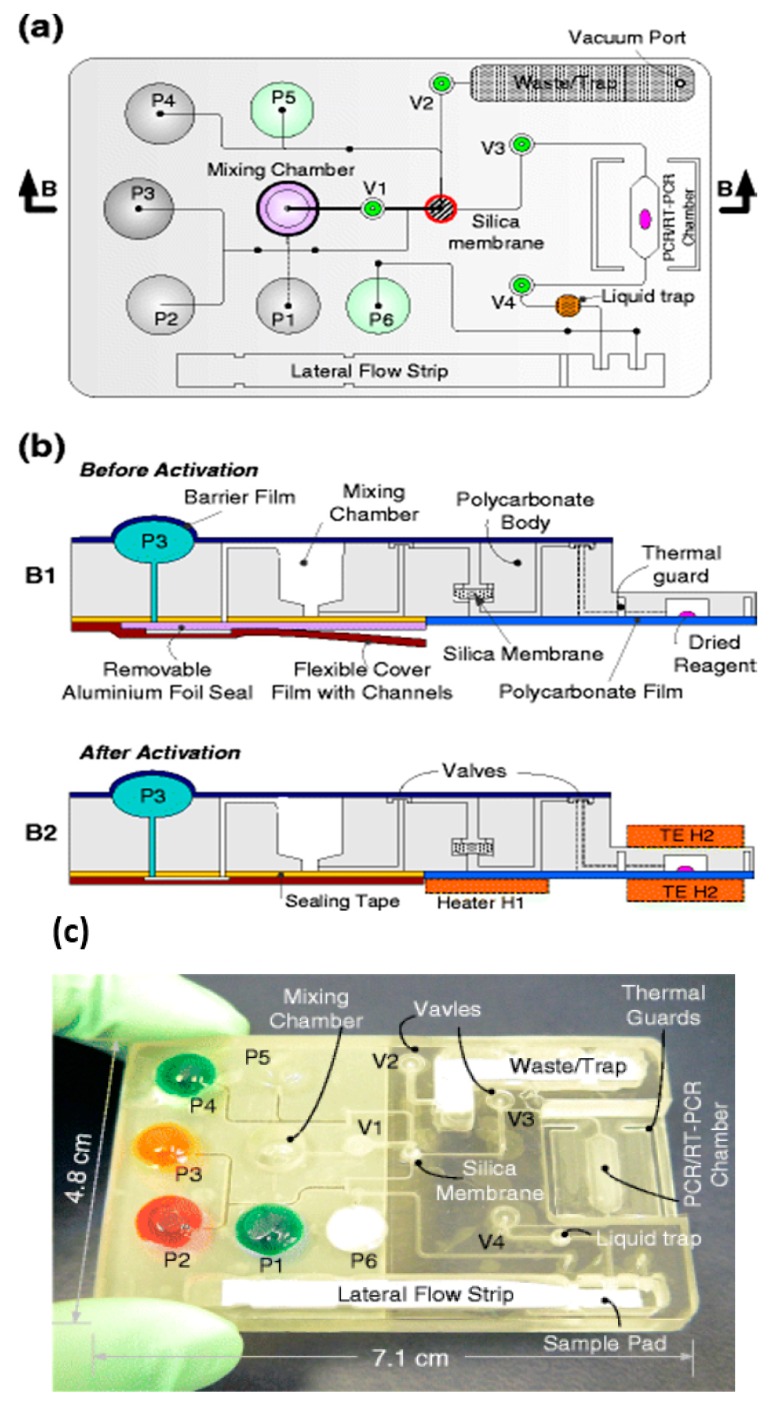
Pouch-based chip integrating sample lysis, solid-phase nucleic acid extraction with a porous silica membrane nucleic acid binding phase, RT-PCR/PCR (reverse transcription polymerase chain reaction) amplification chamber, and lateral flow strip detection of amplicons. Buffer solutions are contained in deformable pouches fabricated into the chip, where depressing the pouch (snap-through) squeezes liquid into channel. Diaphragm valves provide flow control. (**a**) Top plan view and (**b**) cross sections; (**c**) photo of chip. From [52].

The chips in Figure 3, Figure 4 and Figure 5 are made as thin, bonded laminates in acrylic sheet (PMMA) or polycarbonate sheet, but more generally, there is a wide range of materials [83] and fabrication technologies used for microfluidic devices [84,85]. Chips are designed using CAD (Computer-Aided Software) such as SolidWorks™ or AutoCAD™, and prototyped with CO_2_ laser cutting, CNC (computer numerical control) milling, or 3D-printing. The smallest feature size is about 0.1 mm, and channels and chambers have dimensions on the order of 1 mm. Microfabrication technology such as conventional lithography, soft lithography, thin-film deposition, or reactive ion etching are not needed to prototype these devices. The microfluidic circuit (channels, chambers, manifolds) is patterned in a middle layer, channels are enclosed with top and bottom capping layers, sealed by solvent bonding [86], ultrasonic welding [87] thermal-pressure bonding [88], or adhesives, wax bonding [89], and double-sided tapes [90]. Since many adhesives and tapes [91], as well as some chip materials [92,93,94], are known to impede amplification, testing of all chip materials for compatibility with amplification is a must. Incompatibility may be due adsorption of reagents or dyes on surfaces, or chemical inhibition of enzymes. Surface passivation of the amplification chamber is helpful in this regard and can be achieved with BSA and other coatings [95]. In addition, autofluorescence of chip materials may interfere with optical detection [96]. The membrane and encapsulated reagents are inserted during the assembly/bonding. New 3d-printing technologies allow working chips to be prototyped in a few hours [97,98,99,100], so that new designs can be quickly validated and chips can be readily customized for specific diagnostics applications. Designs should be made compatible with injection molding, stamping, or embossing for economical production runs of several hundred (for pre-clinical and clinical trials), and for high-volume manufacturing of tens of thousands of devices for commercialization. The chip prototyping tools (laser machining, CNC mills, 3D printers, thermal bonders) are widely available at educational institutions and machine shops.

## 6. Detection of Amplification

**Figure 6:**
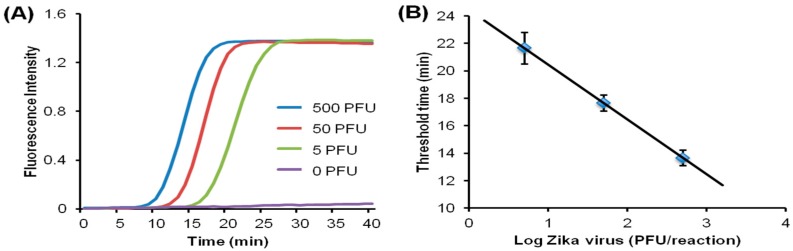
Chip-based LAMP POC diagnostics. Detection with fluorescent intercalating dye: excitation with Smartphone flashlight LED, emission intensity monitored with a Smartphone CCD camera. (**A**) Real-time fluorescence intensity curves as a function of amplification incubation time for four concentrations of Zika virus (0, 5, 50 and 500 PFU, plaque forming units). By establishing a threshold fluorescence level (e.g., time needed for the signal to reach half its saturation value), a threshold time for each concentration can be determined; (**B**) threshold time correlates inversely with the log of Zika concentration (PFU, plaque forming units), providing a calibration curve to estimate Zika concentration in sample. From [103].

**Figure 7:**
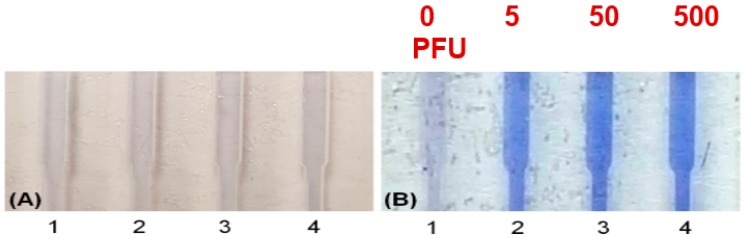
Colorimetric detection reporter for chip-based isothermal amplification. Leuco crystal violet (LCV) changes from colorless to violet in the presence of dsDNA, and offers a simpler visual alternative to fluorescence detection, removing the need for an excitation light, a detector, and filters. Amplification chambers loaded with LCV dye: Starting template concentrations: 0 (NTC, no template control), 5, 50, and 500 PFU (plaque forming units). (**A**) at start of amplification; (**B**) after 40 min. Positive tests exhibit a distinct violet color due to amplicon production. From [103].

**Figure 8:**
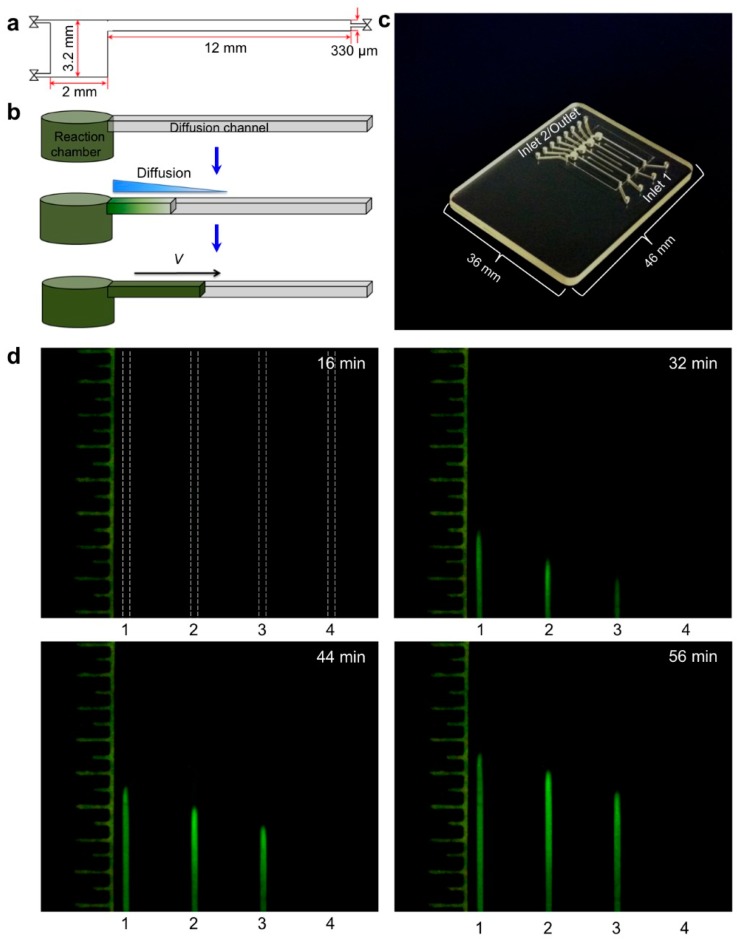

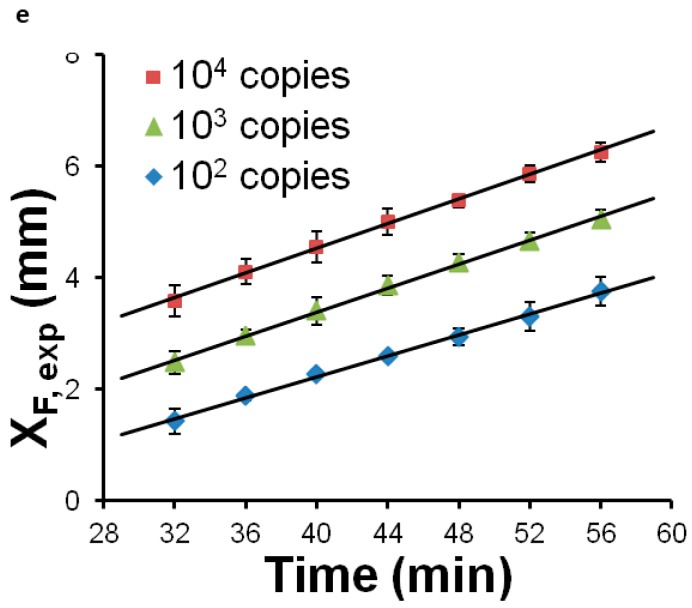
Nuclemeter for Quantifying Nucleic Acids: Nuclemeter chip features an array of reaction-diffusion microconduits for isothermal amplification. The chamber and conduits are filled with LAMP amplification mix including an intercalating DNA green dye, primers, and hydroxypropyl-methyl-cellulose as a low-viscosity sieving matrix to reduce diffusivity. The target nucleic acid template is introduced at the column’s inlet. As isothermal amplification proceeds, the amplification products diffuse down the channel, such that the distance of the reaction front from the chamber *X*_F_ can be correlated with time and template number (copies). At any given time, the length of the fluorescent column can be calibrated against a ruler scale imprinted on the chip. (**a**) Cross-section of reaction-diffusion channel; (**b**) schematic of reaction front, (**c**) photo of nuclemeter chip; (**d**) Photos of reaction diffusion conduits at 0, 16, 32 and 56 min, and (**e**) Diffusion front *X*_F_ position (mm) as a function of time for three different sample (initial) template concentrations: 10^4^, 10^5^ and 10^6^ copies. From [115].

Most POC molecular diagnostics systems use an optical (fluorescence, absorption, luminescence) or visual (color change, turbidity) means of detecting the amplification product. Using a DNA-intercalating dye, such as SYBR™ Green, SYTO™ Green, or Eva Green™, the reaction progress can be monitored by measuring the fluorescence emission intensity [101], excited with ultraviolet or blue light from an LED (Figure 6). Qualitative or semi-quantitative colorometric detection (visible color change of reaction) of amplified dsDNA can be done with dyes such as leuco crystal violet (LCV) [102,103], as shown in Figure 7, pH indicator, or hydroxynaphtol blue [104] added to the reaction. An alternative optical method uses turbidity caused by precipitation products which provides a visual indicator or can quantified by optical absorption measurements [105]. Electrochemical detection methods are also feasible due to the release of protons during polymerase [106,107,108,109,110]. Still another approach features lateral flow strips for the detection of amplicons labeled with conjugated primers, e.g., biotin, that are captured on test line striped with antibodies against the label [111,112,113,114]. Lateral flow strips as such can provide a qualitative (positive/negative) test result. A novel molecular test format (‘nuclemeter’) comprising a reaction-diffusion column provides end-point quantitative detection based on the length of the reacted portion of the column—analogous to temperature measurement from the length of a mercury column in an old style glass capillary thermometer [115]. As shown in Figure 8, template injected into a chamber diffuses along with amplicon through a conduit filled with polymer matrix or made in paper. The conduit is pre-loaded with polymerase, reporter, and primers. As the reaction progresses, a zone of green fluorescence (when fluorescent dye is used) grows, the length of which is proportional to the amount of initial (sample) template and reaction time. A calibration ruler alongside of the channel provides a visual indication of reaction ‘length’ and thus a measure of sample target. In summary, optical methods prove versatile and readily adaptable to POC microfluidics: fluorescence (sensitive, can be quantitative; requires light source, optical filters, and detector/camera), luminescence (sensitive, quantitative, requires detector/camera), nuclemeter (sensitive, simplified quantification, requires light source, filters, and camera) and color change (qualitative, can be read by eye).

## 7. Multiplexing NAATs

**Figure 9:**
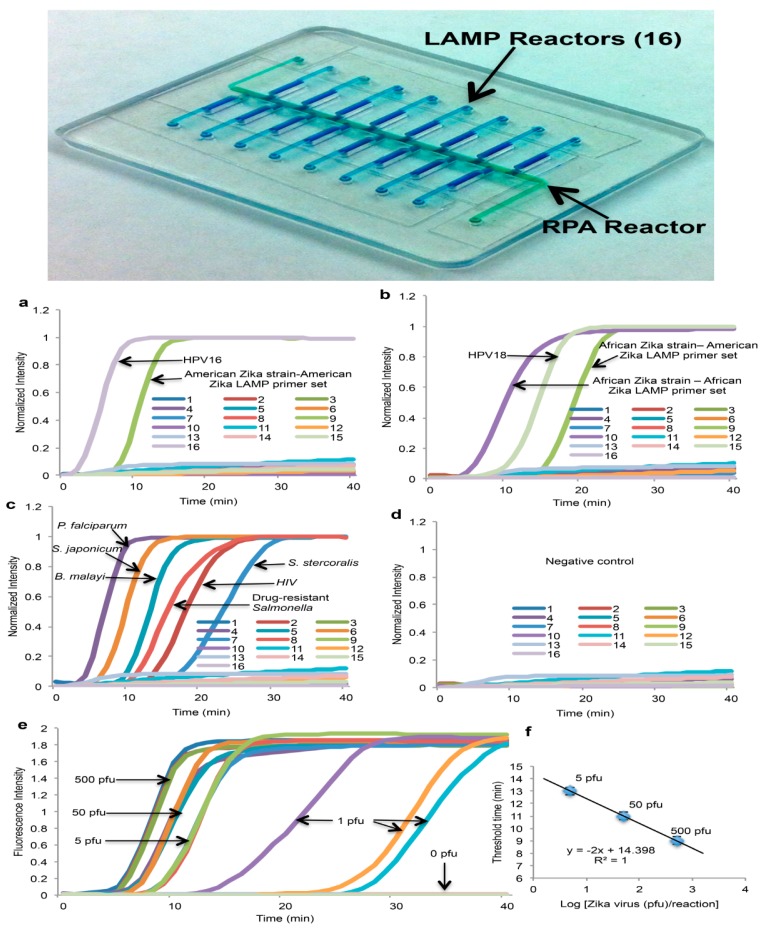
Multiplex two-stage isothermal amplification/detection. Sample is introduced into the center manifold chamber containing primers for up to sixteen different DNA or RNA targets, and amplified with RPA. **Top:** Chip showing center reactor and 16 branching specific LAMP reactors. **Bottom:** Graphs showing real-time fluorescence intensity from sixteen LAMP reaction chambers: (**a**–**c**) Various targets, distributed LAMP chambers; (**d**) negative controls; (**e**) serial dilutions of Zika virus, and (**f**) threshold time vs. Zika template concentration. From [116].

Since many diseases have overlapping symptoms—at least on initial presentation—and require distinct therapies, tests that can discriminate among different pathogens rather than merely confirm the presence of a single pathogen are of considerable interest. In addition, many diseases are coincident, such as co-infections with TB and HIV. Multiplex testing of several targets is possible by providing independent parallel reactors (with appropriate primers) on a single chip, in which case a sample is divided and loaded into individual chambers. A more convenient and better utilization of sample is exemplified by the RAMP (rapid amplification) chip [116], shown in Figure 9. The chip is comprised of a central manifold reaction chamber for the first-stage RPA amplification, and branching capillary reaction chambers for separate, specific second-stage LAMP reactions. The sample is introduced into the central chamber and amplified at 37 °C for 15 min. This reaction contains all primer pairs for simultaneous RPA amplification of all targets of interest. The amplicons from the first reaction diffuse into the branch chambers for LAMP amplification at 63 °C. Each of the branch chambers contains primers for a single target along with enzymes and reporter (Leuco Crystal Violet for colorimetric detection, Eva Green™ fluorescent intercalating dye, or bioluminescent dye). In the implementation shown in Figure 9, 16 separate targets can be concurrently assayed, including Zika, HPV-16, salmonella, HIV, *S. japonicum*, and *P. falciparum*. It should be noted this particular selection of targets has no diagnostics rationale, but instead was chosen based on availability of targets/primers, and as a demonstration of feasibility with combinations of very diverse targets, both RNA and DNA templates, with high sensitivity (in some cases as low as 1 PFU), fast results (<40 min), high specificity (no false positives), ease of use, quantification, and compatibility with minimally processed samples. In summary, multiplexed molecular assays (simultaneous detection of multiple analytical NA targets) can be realized with a parallel array of isothermal NA amplification reactors wherein the isolated NA is distributed to individual reaction chambers containing different pathogen-specific primers. Image capture and analysis (e.g., with a Smartphone CCD camera, see Section 9) can provide detection for multiple reactions through software functions, without added hardware. Such multiplexing provides much-expanded diagnostics capability at a very modest increase in complexity.

## 8. Reagent Stabilization and On-Chip Storage

Chips that contain all reagents, pre-loaded in stabilized form, as well as reservoirs for buffers and other liquids afford simpler designs, more convenient use, and are less susceptible to contamination effects and operator error. The user merely adds the sample to the self-contained chip at the time of use. Flexible, laminated plastic ‘packages’ formed with integral pouch compartments with rupturable seals, actuated by a roller, were described as long ago as 1993 as part of an automated, closed-vessel system for PCR-based diagnostics [117]. This lab-based system was motivated as a means of avoiding false positives from ‘product carryover’ that plagued labs in the early days of PCR, rather than POC applications, but the design concepts are still relevant to contemporary POC NAAT devices.

There are many approaches to microfluidic reagent integration and controlled-release in chips, for both dried reagents and liquids [118]. A self-contained, fully-integrated chip combining sample preparation and PCR wherein reagents were stored in separate compartments, such that they could be hydrated and then mixed with sample and transferred to an amplification reaction chamber, utilized valves fabricated into the chip and relatively complicated flow control [119]. Simpler methods proved feasible including reagent storage in sugar-based matrices [120], glass ampules [121], integrated reagents deposited by inkjet [122], including dried reagents in porous media [123], gelified reagents [124], self-contained gel capillaries [125], dissolvable films [126], microfluidic burst valves [127], and microperforated barrier films [128,129,130].

For POC, LAMP reaction mixes can be lyophilized for long shelf-life (>1 year) and stored in chips during manufacture or inserted, from a library, prior to use. Several protocols for lyophilization of LAMP for microfluidic implementation are available in the literature [131,132,133], and proprietary commercial services that develop customized formulations are available [134]. On-chip pre-storage of buffers and lyophilized reagents is crucial to simplifying operation and providing convenient use of POC diagnostics.

## 9. Smartphone-Based Detection and Electricity-Free Operation

Cellphones are practically universal, even in resource-limited areas of the world not connected to an electrical power grid, and in regions lacking adequate medical infrastructure. Smartphones (cellular phones with operating systems, sensors and cameras, and software applications) have an expanding role in distributed and mobile healthcare, including in vitro diagnostics [135,136,137,138,139,140]. POC devices can be leveraged with cell phones CCD cameras for image capture, smartphone flashlight for fluorescent excitation, and Smartphones to reduce cost, expand functionality, and integrate testing into healthcare networks for data collection, dissemination and reporting, and monitoring epidemics. Smartphones can provide detection using the CCD camera to measure color change or fluorescence intensity from the amplification reaction, as well as add support functions such as adjustment of camera exposure time, computation, user ID, data logging, analysis, communications, and GPS data. Several illustrative examples of Smartphone platforms for POC diagnostics include an immunoassay for STDs (sexually transmitted diseases) [141], a battery-powered, handheld genetic analysis system with PCR microreactors [142], a smartphone-imaged, chip-based reverse-transcription loop-mediated isothermal amplification for HIV detection in whole blood [143], Herpes Simplex Virus (HSV) detection in blood [144], and Zika (ZIKV) in saliva [103].

**Figure 10:**
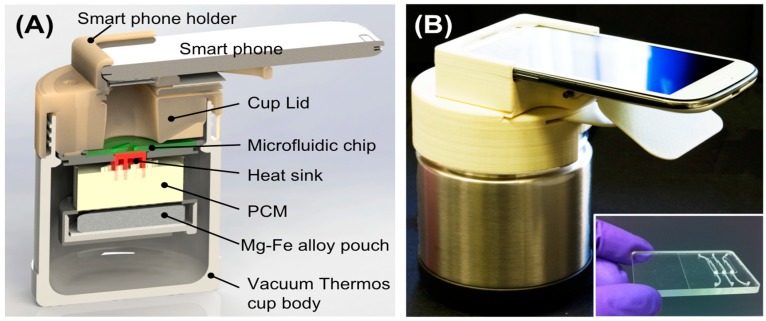
Smart Cup: The Smart cup is a customized Thermos^®^ bottle that provides the inserted single-use chip with constant temperature heating. The Smart Cup has a built in mount for positioning a Smartphone CCD camera to monitor the reaction progress. Heating is due to an exothermic reaction of Mg-Fe powder initiated by addition of water. A phase-change material (PCM) maintains the chip at a constant ~65 °C temperature for about 60 min. (**A**) Cross-section of Smart Cup showing Mg-Fe alloy pouch that is activated by adding water to initiate heating, PCM, heat sink and slot holding microfluidic chip. Smartphone is held for optimal focus of Smartphone CCD camera on chip amplification chamber to measure fluorescence; (**B**) field unit and companion chip (inset). The smart cup can be optionally made of Styrofoam, making the entire system disposable. From [144].

The availability or reliability of electrical grid power can be an issue in many parts of the developing world, and there is an interest in minimally-instrumented POC formats, particularly battery-operated diagnostics devices that can operate free of utilities and generators, or even non-electrical devices that do not need batteries [144,145,146,147,148,149,150,151]. The primary electrical power consumption of a molecular diagnostics device is for controlled incubation of the amplification reaction. Chemical heating, using an exothermic oxidation reaction, offers a means to minimize cell phone battery usage [149,150]. (Electrical consumption of the cellphone is estimated to be 2% of total electrical power needed for electrical heating.) A thermos bottle type chip chemical heating system for use with cellphone detection [144] is shown in Figure 10. In summary, ubiquitous cellphones can be leveraged to provide imaging/detection and other functions to POC systems at little extra cost to the user. The main consumer of electrical energy in POC molecular diagnostics is heating the amplification reaction. Non-electrical heating is feasible to reduce electricity requirements/battery consumption.

## 10. Blood Plasma Extraction Module

The chips described above can directly use plasma or serum, urine, CSF (cerebral spinal fluid), and oral fluid (“saliva”) as samples. Whole blood samples in volumes required for many high sensitivity applications are problematic, due to blood components clogging channels or porous membranes, or blood borne substances such as heme that inhibit enzymatic amplification [152]. In addition, many medical standards are based on viral or bacterial load in cell-free plasma or serum, rather than whole blood. For instance, proviral HIV DNA from white blood cells would confound measurements of HIV viral load interpreted as copies of viral RNA in plasma. Thus, in centralized laboratories, plasma or serum is first separated from whole blood, typically using centrifugation. Centrifugation is neither convenient nor practical for point of care applications, so a miniaturized non-electrical device for relatively large volume plasma extraction is highly useful. Further, FDA Guidelines on CLIA waiver for in vitro diagnostics recommends that simple tests should not rely on centrifugation for sample preparation [153].

There are many examples of blood separation technology for POC applications, as well as reviews and surveys from the perspective of the following criteria: (1) Sample volume accommodated and plasma yield; (2) purity, e.g., concentration of heme in the plasma; (3) prevention of hemolysis, which results in contamination of the plasma with cell components; (4) yield of desired analyte such as virus, bacteria, cell-free nucleic acid, antibodies, and metabolites; (5) whether the blood needs to be first diluted; (6) time for separation (ranging from a few minutes to close to an hour, or as a rate: microliters of plasma produced per minute) [154,155,156,157,158,159,160,161,162]. Most microfluidic plasma extraction devices are limited to very small (~10 μL) volumes of whole blood. Further, for heel or finger prick (capillary) samples, the amount of whole blood available ranges from 0.05 to 0.2 mL, yielding perhaps 20 to 100 μL of plasma, in contrast to venipuncture (needle) blood collection. The suitability of capillary collection for molecular diagnostics is still an issue [163].

**Figure 11:**
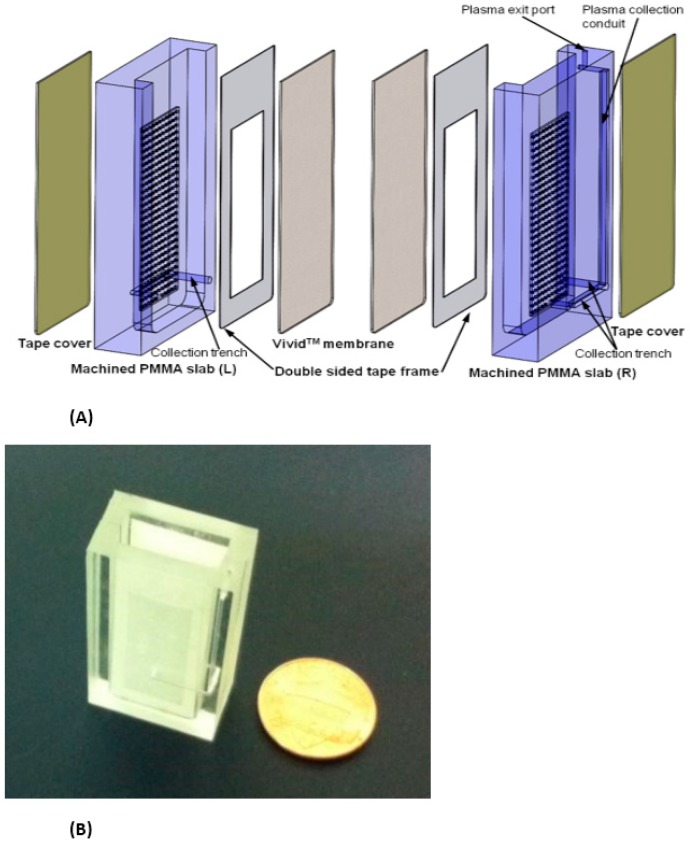
(**A**) Exploded view of pump-free, membrane-based, sedimentation-assisted plasma extraction device. The Vivid™ polysulfone membrane separates plasma from blood cells, and features pores with large (~100 micron) on the whole-blood side that narrow to ~2-micron diameter on the plasma side. (**B**) assembled module. From [163].

**Figure 12:**
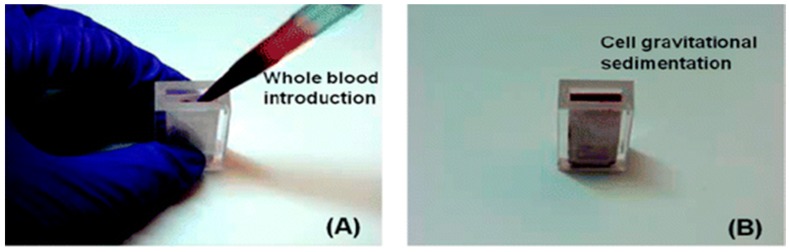

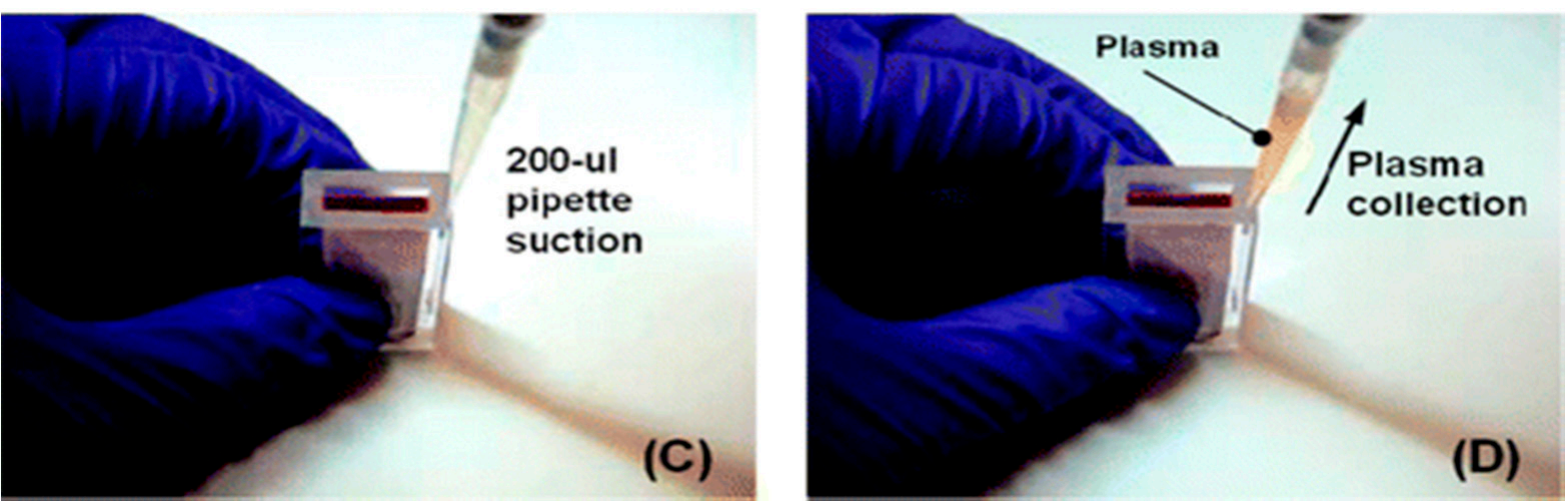
Operation of plasma extraction module. (**A**) Introduction of a 1.8-mL whole blood sample, (**B**) wait 10-min for cell sedimentation, (**C**,**D**), extract 200 μL of plasma. From [164].

**Figure 13:**
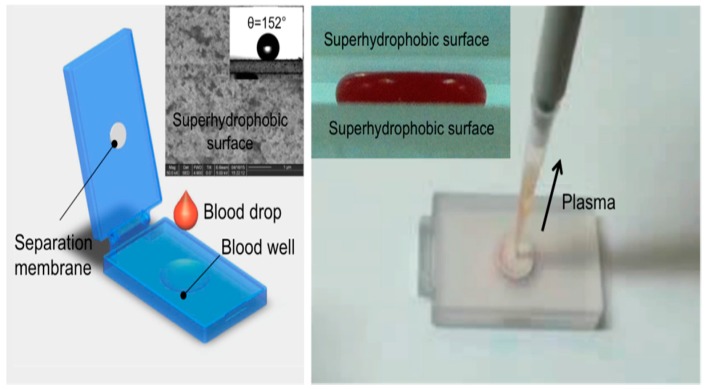
‘Clam-shell’ plasma separator with top mounted filtration membrane for POC applications. **Left**: A drop of blood placed is deposited in well, and lid is closed. The top and bottom inner surfaces have been rendered superhydrophobic by applied coatings to contain the blood drop and discourage analyte adsorption to surfaces. **Inset:** Wetting angle of blood on superhydrophobic surface; **Right:** After ten minutes, allowing for sedimentation of hemocytes, plasma can be aspirated through separation membrane using a pipette. **Inset:** Compressed blood sample between two hydrophobic surfaces. From [165].

Two examples of relatively simple plasma extraction devices that yield high volume of plasma needed for POC NAATs such as HIV viral load without instrumentation are based on membrane filtration and sedimentation. Figure 11 and Figure 12 shows a cartridge type unit [164] and Figure 13 is a clam-shell type device working on similar principles incorporating superhydrophic surfaces [165]. Both can be used at the point of care, and can achieve high sample volume (200 μL), high-yield (~40% of whole blood) plasma extraction for use with NAAT chips. In summary, whole blood samples present a challenge for POC molecular diagnostics due to the need to extract/separate plasma from relatively large sample volumes, without resorting to a centrifugation step. Methods suitable for POC and amenable to integration with chip, using filtration and/or sedimentation, are feasible. 

## 11. Summary, Discussion and Outlook

Lateral flow strip immunoassays and blood glucose monitors, both currently sold over the counter and used at home, could be considered examples of a ‘first generation’ of POC diagnostics devices. Chip-based molecular tests might similarly be regarded as a ‘second generation’ of POC diagnostics. POC microfluidic NAATs utilize non-invasive samples (saliva or urine) or minimally invasive specimens (finger or heel prick blood); integrate sample processing, enzymatic amplification, and detection; and achieve specific and sensitive detection comparable to laboratory-based tests with easy-to-interpret test results facilitated by a smartphone.

POC molecular diagnostics technology can be designed with varying degrees of sophistication with regard to automated versus manual operation, reliance on electrical power, number of parallel tests on a chip, and integration of sample preparation including plasma separation from whole blood. A broad consensus has emerged on design options, converging on solid-phase extraction methods to isolate and concentrate nucleic acids, isothermal amplification, fluorescence optical detection, incorporation of pre-stored, lyophilized reagents and buffers, and smartphone-enabled detection, control, data logging, and communications.

The focus of this review is on minimally-instrumented POC devices relying on pressure-driven fluid actuation and with no valving. Alternate formats include ‘lab on a disk’ systems with fluidic circuits defined in CD-like cartridges that are spun such that centrifugal forces drive fluids through processing stages. For example, a ‘Lab-on-a-Disk’ chip that integrates DNA extraction, isothermal recombinase polymerase amplification (RPA), and lateral flow strip detection, using a laser for cell lysis, actuation of wax valves, and heating, has been described for nucleic acid based testing of foodborne pathogens [166]. Another disk-based microfluidic system with magnetic bead nucleic acid isolation, LAMP, and real-time detection serves as a field-deployable system for molecular detection of malaria parasites [167]. As an example of a POC system somewhat removed from the ultra-simplified, minimally-instrumented systems discussed here is a genomic diagnostics system based on a chip with 60 LAMP reaction chambers, used with a handheld reader featuring LED and phototransistor arrays, waveguides, thin-film heaters mounted on a printed circuit board with microcontroller, thermistor, touch-screen LCD, cellphone transceiver, GPS, USB, and supporting electronics [168]. The porous polydimethylsiloxane (PDMS) chip has a novel method of ‘degas driven’ fluid filling based on vacuum packaging that creates a suction force on exposure to air.

Another format gaining attention in the last decade is based on capillary wicking action in porous materials (‘paper’) similar to that used in lateral flow strips. In such ‘paper microfluidics’ [169,170,171], flow paths are defined by wax printing. Molecular diagnostics tests, including isothermal amplification and colorimetric detection, have been reported. One apparent limitation is the sample volume size that can be accommodated in such devices. Other alternative POC NAAT device formats include chips that with slider mechanisms [172], sometimes combined with capillary wicking action [173,174].

For applications in resource-limited settings, there is interest in minimally-instrumented devices, and particularly ones using chemical heating to avoid reliance on batteries or electricity from utilities or generators. Moreover, the need to process a chip in an instrument can create a bottleneck in the delivery of diagnostics, as instruments that provide temperature-controlled heating, mechanical actuation, and optical detection typically process one or just a few chips at a time with a turn-around time of 30 to 60 min. Thus, for example, an aid worker visiting the village with such a POC diagnostics system could run only ~20 tests in a day.

Computer-aided design, computational fluid dynamics, and rapid prototyping (laser machining, 3d printers) have compressed the design-to-test cycle of chips to a few days or less. Methods for lyophilization of reagents are well established. POC devices should be made of inexpensive plastics and compatible with high-volume production methods such as injection molding, stamping, and ultrasonic welding. For clinical trials, production lots of several thousand microfluidic cassettes can be produced by rapid injection molding companies in a few weeks at a cost of $5 to $10 per device, including cost-of-manufacture, enzymes and other reagents, assembly, and packaging.

The utility and performance potential of POC molecular diagnostics is clear, and the need for fully integrated sample-to-report nucleic acid amplification tests is widely recognized. Nevertheless, alternative technologies may preclude some assumed application areas for molecular diagnostics. The advantages of NAATs over immunoassays may narrow. Improvements in immunoassays stemming from antibody engineering; utilization of nanotechology materials such as quantum dots, up-converting phosphors, fluorophores [175] and surface plasmon resonance reporters; signal amplification, and microfluidic formats [176,177] to reduce the amount of sample, enable additional processing steps such as in consecutive flow assays [178], as well as add multiplexing capability, will enhance the performance of POC immunoassays, while maintaining their cost advantage over NAATs [179].

A second alternative to POC diagnostics is related to sample collection and preservation methods as exemplified by dried blood spot (DBS) technology [180]. In its simplest form, a blood drop collected from a patient is blotted on a filter paper card, dried, and sent to a laboratory where the sample can be reconstituted and analyzed. This approach foregoes rapid test results, but also suggests that complete sample-to-report diagnostics systems need not be at the immediate point of care in order to improve access to healthcare. Similarly, new blood collection tubes with stabilizing agents can preserve blood in liquid form without refrigeration for shipment and storage up to several weeks. Importantly, both DBS cards and collection tubes that preserve liquid blood avoid the need for a cold chain (refrigeration), but sample integrity, especially for liquid biopsies and other methods that require quantitation of biomarkers, is still an issue [181]. Microfluidic-based devices can improve and widen applications of these methods by adding functions such as separating plasma from whole blood, quantifying sample volume, isolating nucleic acids or effecting other separations, adding stabilizers to prevent sample degradation (e.g., of labile RNAs) [182], thus expanding the scope of such approaches in distributed healthcare by offering alternatives to both centralized laboratories and conventional POC devices. A microfluidic system that stores viral RNA extracted from plasma in concentrated form on silica matrix (as used in solid-phase extraction) shows a particular particular implementation for enhanced capabilities in collecting samples in the field [183]. Microfluidic devices may prove useful in providing pre-analytical processing at the point of care for other sample specimen types including stool, CSF, and oral fluid, and for an expanded range of biomarkers that include metabolites, proteins, virus, bacteria, parasites, exosomes [184], and cell-free nucleic acids [185,186,187] including microRNAs [188].

Speculating on formats and applications of POC molecular diagnostics in the next decade, in view of the many different purposes, venues of use, sample types and sizes, volume and frequency of testing, and sophistication of user, suggests a segmented market with sometimes difficult-to-predict commercial potential. Devices for home-use, sold over the counter (OTC), would ideally be non-instrumented (other than cellphone detection), and as easy to use as lateral flow strips currently sold in drug stores. Here, paper microfluidics with a low-temperature NA amplification (e.g., RPA at 37 to 42 °C) may have an edge. Manually-actuated microfluidic chips made in hard plastic with integrated pouches for fluid storage and actuation, and possibly incorporating chemical heating, can provide convenient quantification of target, such as for HIV viral load testing. This type of POC system may also serve non-diagnostics applications ranging from testing for food and water contamination, genotyping, and biodefense. More complicated systems, perhaps for doctor and dentist offices, pharmacies, and store-front clinics, would utilize more complicated table-top support instrumentation with electro-mechanical actuators for automated operation and interfacing with a laptop computer. Such a platform could be readily programmed to operate with different chips as needed, well as chips for simultaneous multiplex detection of several pathogens and biomarkers.

## 12. Conclusions

Point-of-care molecular diagnostics technology, or more specifically microfluidic nucleic acid amplification tests (NAATs), can be realized in a variety of formats from simple manually operated, or minimally instrumented formats appropriate for resource-limited settings, to chips that operate with benchtop instruments that provide fluid actuation, flow control, reagent delivery, temperature-controlled heating/cooling, and optical detection, for use in remote or mobile laboratories. The ‘back end’ of the chip, including solid-phase nucleic acid isolation and concentration, enzymatic amplification—most simply with isothermal reactions—and end-point or real-time detection, often utilizing a cell phone, is well established and sufficiently generic for adaptation to many applications of infectious disease tests. However, the ‘front end’ of the chip, including interfacing with a sample collector, plasma extraction from blood samples, filtering or dilution of sample, lysis, and stabilization (e.g., inactivation of nucleases) is dependent on sample type (e.g., blood, urine, saliva) and volume (10 to 1000 μL), technical skills of users is user A wide range of sample storage (lyophilization) and liquid storage, including blisters or pouches integrated into the chip, are feasible. There appears to be no shortage of ideas regarding flow control and fluid actuation, but no consensus on implementation either. Further, most applications of POC diagnostics are directed toward detecting biomarkers in blood, which generally implies a need for extracting plasma from blood, often in relatively large volumes (>100 μL) in venous blood draws, in contrast to capillary draws with finger or heel prick samples (10 to 20 μL). The seamless integration of plasma extraction capability with molecular diagnostics has proved challenging. This review focused on simple, microfluidic chips that integrate NA isolation, on-chip dry-stored reagents, isothermal amplification, and cellphone-based detection that can be operated by manual pipetting, or with addition of pouches or blister packs, in a more autonomous mode. A few examples of plasma separation modules with large volume capacity (>200 μL) were described.

## Figures and Tables

**Table 1:** Performance and features objectives for point-of-care (POC) nucleic acid amplification tests.

Criteria	Aim	Comments
Test Cost	$5–10 consumables per test	Disposable, single-use plastic cartridge, pre-loaded with reagents Compatible with injection molding,
Instrument Cost	<$500	Portable, handheld or desktop, providing temperature regulation, stable optical platform for cellphone or other detector, limited actuation; made with off-the-shelf components
Performance: Sensitivity, Limit of Detection (LOD); Specificity	Comparable to laboratory tests	e.g., HIV viral load testing LOD: 1–100 virons/mL plasma; Screening tests LOD: 1000 genome copies per sample. Sensitivity/Specificity (false negatives/ false positives): 98 to 99%.
Time for testing	30 to 60 min	Time spanning sample-in to report-out should be about 30 min, primarily due to time required for amplification of low-concentration target
Operator skill level	minimal, non-professional, semi-skilled	Training in less than 1 h. No pipetting, sample collection and loading raw sample into cartridge, no sample transfer after sample loading (e.g., no pre-sample processing with centrifuge), no addition of reagents at time of use.
Power	battery or chemical heating/cellphone	Ideally, should operate independent of grid electric power (Many resource-limited areas of the world do not have reliable electric power.)
Shelf life	test cartridge: 1 year @ 40 °C	No cold-chain, hermetically sealed cartridge can be stored in tropical climates for a year without refrigeration.

**Table 2:** Design options for microfluidic implementation of nucleic acid amplification test unit operations.

Unit Operation	Options	POC Issues
Blood Fractionation	centrifugation filtration sedimentation fluid-flow fractionation	Centrifugation is difficult to integrate into the chip, and a separate centrifugation step may be needed. Most LOC filtration/sedimentation and fluid flow fractionation is typically limited to small sample volumes.
Lysis	mechanical chemical enzymatic thermal	Viruses are comparatively easy to lyse with detergents and chaotropic salts; vegetative bacterial cells are somewhat more difficult; and spores most difficult, requiring enzymes and/or mechanical disruption.
Nucleic Acid (NA) Isolation (Extraction, Purification, Concentration)	solid-phase extraction liquid-liquid extraction ethanol precipitation hybridization (e.g., magnetic beads) centrifugation	Solid-phase extraction using a NA-binding phase (e.g., silica) and binding, wash, and elution buffers is readily implemented with microfluidics.
Nucleic Acid (NA) Amplification	PCR (polymerase chain reaction) isothermal methods (e.g., AMP, RPA, HDA, NASBA)	PCR is well developed, but requires instrumentation for precise thermal cycling, and has relatively high power consumption. Isothermal methods require much less instrumentation. LAMP (65 °C constant temperature incubation) appears to be the most used method.
Amplicon Detection	fluorescent dyes bioluminescent reporters colorimetric dyes electrochemical sensors	Fluorescent dyes are very sensitive. Bioluminescent reporters do not require light sources or optical filters. Colorimetric dyes can be read by eye for instrument-free operation. Electrochemical sensors are more compact, but generally less sensitive and difficult to interface with a disposable chip.
